# Sex-Specific Neural Networks of Cued Threat Conditioning: A Pilot Study

**DOI:** 10.3389/fnsys.2022.832484

**Published:** 2022-05-17

**Authors:** Kamryn C. du Plessis, Sreetama Basu, Timothy H. Rumbell, Elizabeth K. Lucas

**Affiliations:** ^1^Department of Molecular Biomedical Sciences, College of Veterinary Medicine, North Carolina State University, Raleigh, NC, United States; ^2^Department of Neurosciences, Cleveland Clinic, Cleveland, OH, United States; ^3^IBM Thomas J. Watson Research Center, Yorktown Heights, NY, United States

**Keywords:** fear conditioning, classical conditioning, associative memory, memory encoding, immediate early gene, functional connectivity, graph theory, negative valence systems

## Abstract

Cued threat conditioning is the most common preclinical model for emotional memory, which is dysregulated in anxiety disorders and post-traumatic stress disorder. Though women are twice as likely as men to develop these disorders, current knowledge of threat conditioning networks was established by studies that excluded female subjects. For unbiased investigation of sex differences in these networks, we quantified the neural activity marker c-fos across 112 brain regions in adult male and female mice after cued threat conditioning compared to naïve controls. We found that trained females engaged prelimbic cortex, lateral amygdala, cortical amygdala, dorsal peduncular cortex, and subparafasicular nucleus more than, and subparaventricular zone less than, trained males. To explore how these sex differences in regional activity impact the global network, we generated interregional cross-correlations of c-fos expression to identify regions that were co-active during conditioning and performed hub analyses to identify regional control centers within each neural network. These exploratory graph theory-derived analyses revealed sex differences in the functional coordination of the threat conditioning network as well as distinct hub regions between trained males and females. Hub identification across multiple networks constructed by sequentially pruning the least reliable connections revealed globus pallidus and ventral lateral septum as the most robust hubs for trained males and females, respectively. While low sample size and lack of non-associative controls are major limitations, these findings provide preliminary evidence of sex differences in the individual circuit components and broader global networks of threat conditioning that may confer female vulnerability to fear-based psychiatric disease.

## Introduction

Women are twice as likely as men to experience a psychiatric disease characterized by dysregulation of emotional memory, including post-traumatic stress disorder, generalized anxiety disorder, panic disorder, and phobia (Altemus et al., 2014; Pittig et al., 2018). Cued threat (or fear; Mobbs et al., 2019) conditioning is the most widespread preclinical model of emotional memory (Milad and Quirk, 2012). In this paradigm, a neutral conditioned stimulus (CS; i.e., auditory tone) is paired with an innately aversive unconditioned stimulus (US; i.e., footshock). Subjects form an associative memory between the two stimuli and subsequently express acute threat responses to the predictive CS. Decades of threat conditioning research have uncovered molecular, cellular, and circuit-based mechanisms driving threat memory encoding (Maren, 2001; Herry and Johansen, 2014; Fanselow and Wassum, 2015). However, despite increased susceptibility to disorders of emotional memory, more than 98% of these studies excluded female subjects (Lebron-Milad and Milad, 2012).

To address this gap in knowledge, this pilot study assessed functional connectivity through brain-wide quantification of the neural activity marker c-fos followed by graph theoretical analyses to explore sex differences in the neural networks recruited by cued threat conditioning. Synaptic plasticity amongst neurons engaged by threat conditioning creates the anatomical infrastructure required for later threat memory recall (Josselyn et al., 2015). Thus, the organizing influence of activity during acquisition is a logical starting point for investigating female threat memory processes. These processes engage broadly distributed functional networks consisting of integrated clusters of regions with strongly correlated activity (Park and Friston, 2013). While *in vivo* techniques are often used to assess functional connectivity, analysis of covariance in activity-dependent immediate early gene (IEG) induction is a comparable technique in postmortem tissue (Wheeler et al., 2013; Vetere et al., 2017; Rogers-Carter et al., 2018; Ben-Ami Bartal et al., 2021). Here, we captured brain-wide expression of the IEG c-fos during cued threat conditioning and constructed unbiased exploratory maps of male and female threat conditioning networks.

## Results

### No Behavioral Sex Differences During Classical Threat Conditioning

**FIGURE 1:**
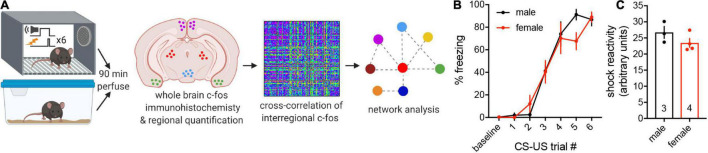
Experimental pipeline and behavioral quantification. **(A)** Adult male and female mice were randomly assigned to cued threat conditioning or naïve homecage conditions. Mice were perfused 90 min later, and brain-wide neuronal activation was assessed through immunofluorescence staining and quantification of the immediate early gene c-fos. Interregional correlations of c-fos expression were computed across 112 brain regions to determine functional connectivity. Supra-threshold correlations were then used to generate functional networks and identify hub regions for threat conditioning using graph theoretical approaches. Schema made with Biorender.com. **(B)** No differences in freezing during the CS were observed between trained males and females across CS-US pairings during threat conditioning. **(C)** No differences in average shock reactivity were observed between males and females during threat conditioning. Data presented as mean ± SEM. **(B)** Two-way repeated-measures ANOVA. **(C)** Two-tailed *t*-test. n/group denoted on bar histogram in panel **(C)**.

Adult male and female mice were randomly assigned to a cued threat conditioning paradigm (trained group, *n* = 3–4) or to remain in their homecages (naïve control group, *n* = 5; Figure 1A). We measured freezing, the dominant defensive response evoked by threatening stimuli (Blanchard and Blanchard, 1969; Fanselow, 1980), during the CS across CS-US pairings to assess threat memory acquisition. Two-way repeated-measures ANOVA revealed a main effect of CS-US pairing [*F*_(6, 30)_ = 78.99, *p* < 0.0001] but no main effect of sex (*p* = 0.70) and no interaction (*p* = 0.26), indicating that males and females acquire conditioned threat memories at the same rate (Figure 1B). Freezing during the inter-trial intervals also did not differ between the sexes (Supplementary Figure 1A). As sex differences in passive vs. active conditioned threat responses have been reported (Gruene et al., 2015), we next quantified instances of darting and found no evidence of sex differences in conditioned flight behavior (Supplementary Figure 1B), similar to previous investigations in C57Bl/6J mice (Borkar et al., 2020; Florido et al., 2021; Taylor et al., 2021). Finally, we observed no sex differences in average shock reactivity (two-tailed *t*-test, *p* = 0.21; Figure 1C) or shock reactivity across CS-US trials (Supplementary Figure 1C). Together, these findings demonstrate that any sex differences in regional or interregional activity revealed in subsequent analyses would be unlikely to be due to differences in behavioral responses during threat conditioning.

### Cued Threat Conditioning Induces Limited Sex Differences in Regional c-fos Expression

**FIGURE 2:**
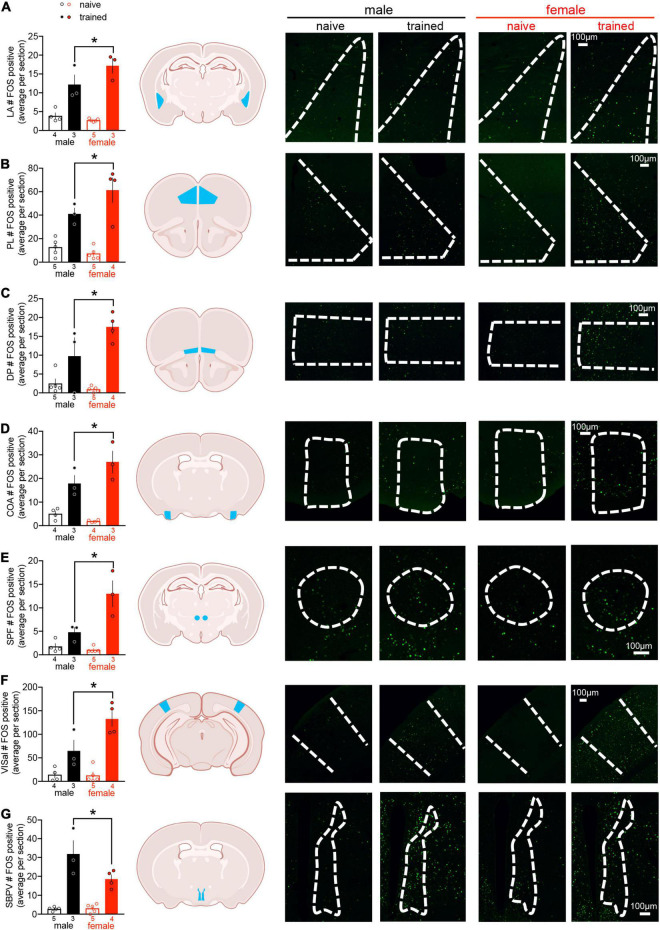
Cued threat conditioning induces limited sex differences in regional c-fos expression. Quantification of regional c-fos expression revealed sex differences in trained males compared to trained females in 7/112 regions: **(A)** lateral amygdala (LA), **(B)** prelimbic cortex (PL), **(C)** dorsal peduncular cortex (DP), **(D)** cortical amygdalar area (COA), **(E)** subparafascicular nucleus (SPF), **(F)** anterolateral visual area (VISal), and **(G)** subparaventricular zone (SBPV). Data presented as mean ± SEM are on the left. Schemas with each region denoted in blue are in the middle (made with Biorender.com). Representative confocal images with regions outlined in white dashes are on the right. The scale bar on the far right confocal image represents all images for a given brain region. For all regions, two-way ANOVA revealed a main effect of training (*p* < 0.05) and an interaction between training and sex (*p* < 0.05). Planned *post hoc* comparisons between trained males and females were conducted with Fisher’s LSD, *p < 0.05. For statistical details, see Supplementary Table 2. n/group denoted under bar histograms.

Action potential generation is associated with rapid *de novo* transcription and translation of the IEG c-fos (Morgan et al., 1987). To investigate neural networks associated with threat conditioning in males vs. females, c-fos protein expression was quantified as a proxy for neuronal activation across 112 brain regions (for a list of regions and their abbreviations, see Supplementary Table 1; for representative microscopy, see Supplementary Figure 2). Two-way ANOVA of regional cell counts revealed a main effect of training in 93 brain regions, indicating that only a small subset of regions were not recruited by threat conditioning (for statistics, see Supplementary Table 2). In addition to a main effect of training, we identified a main effect of sex in the AIv, CLA, SBPV, SPF, and VISal (Supplementary Table 2). In the SPF and VISal, c-fos counts were greater in females compared to males, whereas males displayed greater c-fos expression than females in the AIv, CLA, and SBPV. Finally, we found interactions between sex and training in CEA, COA, DP, LA, PL, SBPV, SPF, and VISal (Supplementary Table 2). To better interpret these interactions, we conducted planned *post hoc* comparisons and observed differences between trained males and trained females in all regions except CEA. Trained females exhibited greater c-fos expression than trained males in all regions except SBPV, where the opposite effect was observed (Figure 2). These data demonstrate sex differences in regional activation of well-studied (LA, PL) and potentially novel (COA, DP, SBPV, SPF, VISal) mediators of threat conditioning.

### Striking Sex Differences in Functional Connectivity

**FIGURE 3:**
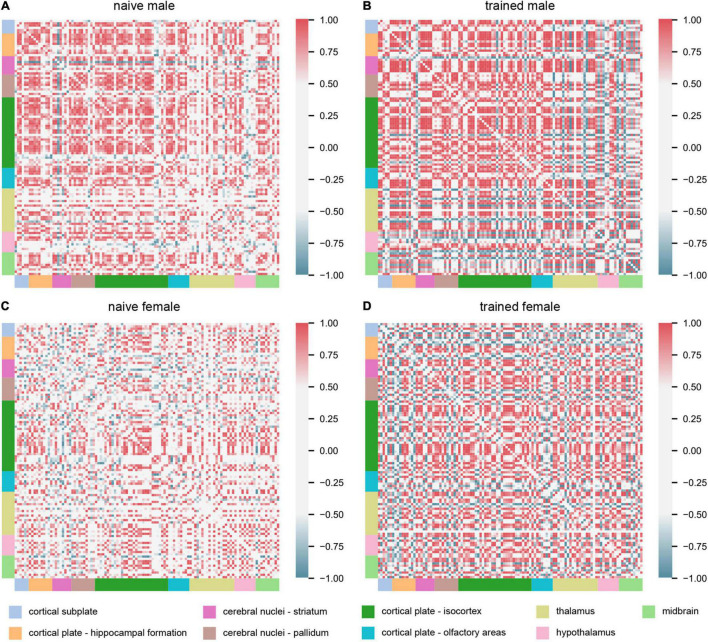
Cued threat conditioning engages broadly distributed, sex-specific functional networks. Matrices showing interregional correlations of c-fos expression across experimental groups: **(A)** naïve male (*n* = 4), **(B)** trained male (*n* = 3), **(C)** naïve female (*n* = 4), and **(D)** trained female (*n* = 3). The axes correspond to 112 brain regions organized into major divisions, represented by colored bars. For an ordered list of brain regions represented in the matrices, see Supplementary Table 3. Matrix colors represent the strength of the correlation (Pearson’s *r*; scale, right).

Interrogation of interregional c-fos expression enables detection of covariance in activation and therefore functional connectivity between two brain regions (Horwitz et al., 1995; Park and Friston, 2013). We computed Pearson’s correlation coefficients of interregional c-fos expression across experimental groups (Figure 3). These analyses should be considered exploratory, as we only compared animals with matched representation of all 112 brain regions under investigation, resulting in an *n* = 4 for the naïve groups and *n* = 3 for the trained groups. We first made the observation that naïve males exhibited greater positive interregional correlations than naïve females, indicating that sex differences in neural network coordination exist independent of training. We next observed distinct patterns of network coordination between trained groups. In trained males, several groups of regions were co-activated with most other regions. However, the same regions in trained females showed much greater variation in their coordination relative to the rest of the network. Thus, despite the limited sex differences observed in regional activation (Figure 2), striking qualitative sex differences were observed for the broader threat conditioning network (Figure 3).

**FIGURE 4:**
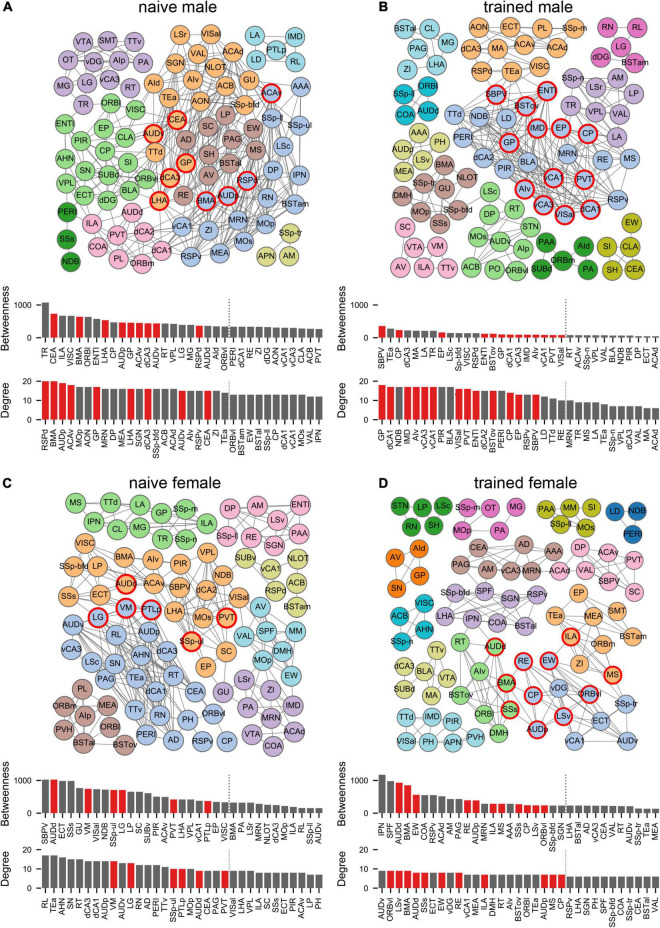
Males and females recruit distinct network structures for cued threat conditioning. Network graphs showing brain regions (nodes) as circles and supra-threshold correlations between regions (edges) as lines for **(A)** naïve male, **(B)** trained male, **(C)** naïve female, and **(D)** trained female groups. Node colors correspond to clusters found using the Markov clustering algorithm applied to the adjacency matrix for each group, which identifies sets of nodes that are more connected with each other than with nodes outside of the cluster. Bar plots show centrality measures of degree (number of edges) and betweenness (number of shortest paths through a node). The top 30% of nodes for each measure are shown. Hubs (red) are nodes within the top 20% (indicated by the vertical dotted line) for both measures. See Supplementary Table 1 for a list of regions and their acronyms.

To quantify these network differences, we next interrogated the correlation matrices with graph-theoretic measures to explore the structure of threat conditioning networks and identify regions exerting considerable influence over network coordination (Wheeler et al., 2013; Vetere et al., 2017; Rogers-Carter et al., 2018; Ben-Ami Bartal et al., 2021). We thresholded each correlation matrix to retain only statistically significant (*p* < 0.05) positive connections between regions to create binary, undirected network graphs (Figure 4). To aid network visualization, Markov clustering was used to identify clusters of interconnected nodes (brain regions), whereby regions are more likely to be correlated with other regions within their cluster than to regions in other clusters (Wheeler et al., 2013). Within these functional networks, coordinated activity may be disproportionately influenced by activity in brain regions at prominent locations in the network structure (Vetere et al., 2017). To identify such ‘hub’ regions, we computed two measures of node centrality: (1) *degree* represents the number of connections to a node and (2) *betweenness* represents the fraction of shortest paths through the network that include a node. Together, these measures identify nodes that are interconnected with many neighbors and lie on many paths through the network. Nodes that ranked in the top 20% for both measures were considered hubs (Wheeler et al., 2013; Ben-Ami Bartal et al., 2021). Regardless of experimental group, identified hub nodes tended to belong to larger clusters and often bordered other large clusters (Figure 4). Interestingly, hubs were largely non-overlapping between groups with no hubs shared between naïve males and females and a single hub (CP) shared between trained males and females. Importantly, despite the segregation of hubs between the sexes, canonical mediators of innate and learned threat were over-represented as hubs for both males (BSTov, PVT, ENTl, vCA1, vCA3, dCA1, AIv) and females (BMA, ILA, LSv, RE, MS, AUDd, AUDp, SSs).

Given the low *n* per group, a high false positive rate may exist among our thresholded connections. Therefore, we generated 1000 random networks from each data-generated network by shuffling the connections present in the data-generated network while maintaining the same number of active nodes, edges per node, and degree distribution (Rubinov and Sporns, 2010). We then computed three network-level measures on the data-generated and random networks to determine whether data-generated network properties were likely to emerge from networks generated through random selection of false positive correlations: (1) *transitivity* to measure network segregation (Newman, 2003), (2) *assortativity* to measure the correlation between degrees of nodes (Newman, 2002), and (3) *small-worldness* to measure small-world structure (Humphries and Gurney, 2008; see Supplementary Methods for details). In data-generated networks, these measures demonstrated substantial structure that was absent in random networks (Supplementary Figure 3), with high transitivity and assortativity mirroring values reported in similar analyses (Wheeler et al., 2013). Notably, data-generated networks also had a higher level of small-world structure than random networks. Small-world structure, characterized by large clusters of nodes with short paths between clusters, is associated with efficient communication in complex brain networks (Bullmore and Sporns, 2012) and has often been demonstrated in studies of brain network anatomy and function (Park and Friston, 2013). Thus, despite the low sample size, the network properties present in the data-generated networks cannot be replicated in random control networks.

As network properties may vary substantially based on the parameters used for construction, we generated network graphs based on 20 different *p*-value thresholds (from *p* < 0.005 to *p* < 0.1) to check whether the network structure we found was dependent upon our specific choice of threshold (e.g., Wheeler et al., 2013). We generated 1000 random graphs at each *p*-value threshold, as described above. Measures of transitivity, assortativity, and small-worldness were reasonably stable across all *p*-value thresholds, and random networks always had lower values for all measures across all thresholds (Supplementary Figure 4). These findings indicate that the presence of greater structure than would be expected in a comparable random network was not dependent on the specific *p*-value threshold we chose.

Finally, hub region identity may be influenced by small changes in the connections included in a data-generated network. Therefore, we computed hub regions for 9 additional network graphs per group using lower *p*-values for thresholding (from *p* < 0.005 to *p* < 0.045) to prune connections and confirm the identity of regions that reliably emerge as network hubs across varying threshold parameters (Supplementary Figure 5). Based on these analyses, GP emerged as the most robust hub for trained males, whereas LSv emerged as the most robust hub for trained females.

## Discussion

This pilot study investigated the neural networks of cued threat conditioning in male vs. female mice using brain-wide c-fos quantification and graph theory-derived analyses. Similar to a prior investigation of whole-brain IEG expression after cued threat conditioning in male mice (Cho et al., 2017), we find that training engages most brain regions. We demonstrate sex differences in both regional activation and functional network structure in response to cued threat conditioning. Trained females recruited COA, DP, LA, PL, SPF, and VISal to a greater extent than males, whereas SBPV was the only region in which we observed higher activation in trained males compared to females. In subsequent analyses, we used interregional c-fos covariance to discern functional connectivity within the male and female threat conditioning networks. Exploratory graph theoretical analyses identified hub regions exerting disproportionate influence over coordination of these networks with very limited overlap between experimental groups. While insufficient sample size is a major limitation, network organization and structure present in all experimental groups were unable to be replicated through generation of random control networks. Together, these data provide preliminary evidence of sex differences in neural networks underlying threat conditioning.

We report training-dependent sex differences in activation of two widely studied regions of the threat memory circuit: LA and PL. During auditory threat conditioning, sensory information conveying the CS and US converge in LA, a primary site of associative plasticity required for threat memory formation (Nabavi et al., 2014; Sears et al., 2014; Kim and Cho, 2017). Similarly, PL also undergoes experience-dependent plasticity during threat memory encoding and mediates memory recall (Arruda-Carvalho and Clem, 2015). We observed increased activation of both LA and PL in trained females vs. males. Interestingly, previous work has shown increased baseline excitatory input and learning-dependent synaptic plasticity in LA of females compared to males (Chen et al., 2014; Blume et al., 2017). Sex differences in the activation of PL neurons and their role in threat memory recall and persistence have also been reported (Fenton et al., 2014; Giannotti et al., 2019). Further experiments are required to determine the causal relationship between enhanced activation of LA/PL and threat memory processes in females.

We also identified enhanced activation of potentially novel mediators of threat conditioning in females compared to males: COA, DP, SPF, and VISal. COA, a chemosensory amygdala region, receives direct projections from the main and accessory olfactory bulbs and exhibits experience-dependent plasticity following olfactory threat conditioning (Swanson and Petrovich, 1998; Sevelinges et al., 2004). DP is an olfactory division of the medial prefrontal cortex shown to mediate sympathetic stress responses (Luskin and Price, 1983; Kataoka et al., 2020). Thus, while behavioral sex differences were not observed, increased recruitment of DP in females could drive sex differences in threat-evoked sympathetic output. Interestingly, COA is unidirectionally (Cadiz-Moretti et al., 2017) and DP bidirectionally (Wang et al., 2006a,b) connected to SPF, a thalamic region proposed to relay auditory and visual information to LA during threat conditioning (LeDoux et al., 1985; Romanski and LeDoux, 1992; Coolen et al., 2003; Lanuza et al., 2004). Further experiments are required to determine if enhanced activation of these interconnected brain regions is associated with a novel, multimodal sensory circuit causally linked to threat memory acquisition in females.

SBPV was the only region in which we observed greater activation in trained males vs. females. SBPV regulates circadian control of aggressive behavior in male mice through inhibitory projections to the hypothalamus (Todd et al., 2018). As predator-induced threat suppresses aggressive behaviors (Blanchard et al., 1984), increased SBPV activation in trained males could reflect heightened inhibition of aggression in favor of competing defensive behaviors such as freezing.

Expanding upon these regional sex differences, our interregional network analyses revealed strikingly distinct functional networks between both naïve and trained males and females. Notably, of the 24 network hub regions identified, CP was the only common hub between males and females, highlighting near total segregation of threat conditioning networks between sexes. While many canonical mediators of innate and learned threat were identified as hubs, GP emerged as the most robust hub for males whereas LSv emerged as the most robust hub for females. While not historically considered in investigations of threat memory, a recent study in male and female mice found that CEA projections to GP encode the US and are both necessary and sufficient for threat memory formation (Giovanniello et al., 2020). Unfortunately, no comparisons between the sexes were reported. The LS is a sexually dimorphic hub of the limbic system (Sheehan et al., 2004). In males LSv encodes aversive stimuli and drives defensive behavioral responses (Mongeau et al., 2003; Xu et al., 2019; Mu et al., 2020), but a specific role for the LSv in classical threat conditioning has not been elucidated in either sex. Our findings warrant further investigation of the roles of GP and LSv in threat memory dynamics.

Intriguingly, these sex differences in neural activity and network structure occurred in the absence of behavioral differences. According to the dual-function hypothesis, neural sex differences may serve as compensatory mechanisms through which physiological differences between males and females are overcome to drive equivalent behavioral phenotypes (De Vries, 2004). Thus, the observed sex differences in regional activation and functional networks may be necessary for similar expression of conditioned defensive behaviors between males and females during training. On the other hand, neuronal ensembles active during memory encoding undergo plasticity during consolidation to allow for their re-engagement during memory retrieval (Josselyn et al., 2015). Therefore, the identified neural sex differences may reflect the priming of sex-specific circuits for memory recall. While not assessed in the current study, sex differences in threat memory recall, though often conflicting, have been reported (Shansky, 2015).

In addition to low sample size, several limitations in the overall design of the present study should be highlighted. First, our experiment only considered naïve animals as a control group. Therefore, any aspect of the threat conditioning procedure could have induced c-fos expression, including handling, context exploration, CS exposure, and/or US exposure. While additional controls such as tone-alone, shock-alone, and unpaired groups are commonly employed in investigations of cued memory recall, interpretation of such control groups in the investigation of IEG expression following training is more complex, as individual neurons must respond to multiple stimuli to encode the association between them. Likely owing to this interpretational complexity, very few studies have investigated IEG expression post-training (Radwanska et al., 2002; Ploski et al., 2008; Lonergan et al., 2010; Lelos and Good, 2012; Peter et al., 2012; Cho et al., 2017; Li et al., 2019; Ivashkina et al., 2021). Among these, Ploski et al. (2008) observed increased c-fos expression between trained and control groups in LA; importantly, naïve, tone-alone, and shock-alone control groups did not differ. However, Cho et al. (2017) reported highly overlapping brain-wide IEG expression between training and tone-/shock-alone control groups. The temporal resolution of *in vivo* techniques (i.e., electrophysiology, calcium imaging) is required to disentangle neuronal responses to discrete stimuli during training, and *in vivo* manipulation (i.e., optogenetics, chemogenetics) is required to assess the causal relationship between the observed neuronal activity and memory formation. Second, we did not account for estrous cycle stage. While consideration of estrous cycle is not necessary for sex differences research (Prendergast et al., 2014), both estradiol and progesterone have been shown to modulate limbic system activity and threat memory processes in both rodents and humans (Lebron-Milad and Milad, 2012). Follow-up studies should determine if neural networks for threat conditioning vary across the estrous cycle. Finally, although c-fos expression is widely used as a marker of neuronal activity (Morgan et al., 1987), limitations exist (Kovacs, 2008). Action potential firing alone is not sufficient for c-fos expression in some neurons (Luckman et al., 1994), and differential DNA methylation patterns between cell types bias c-fos expression (Mo et al., 2015). Quantifying other IEGs such as Arc or Egr-1 in conjunction with c-fos may be helpful in validating our findings in future studies.

In conclusion, this pilot study provides an initial glimpse into the previously uncharted female threat conditioning network and offers rich datasets for future investigation. While regional differences in neuronal activation were limited, exploratory network analysis revealed striking sex differences in the broader neural networks engaged by threat conditioning. Ultimately, these preliminary analyses emphasize the value of unbiased network-scale inquiry of memory processes and provide the foundations for future translational studies.

## Materials and Methods

Further methodological details can be found in Supplementary Material.

### Animals

All experiments were approved in advance by the Institutional Animal Care and Use Committee at NC State University. Adult (8–12 weeks) male and female C57BL/6J mice (Jackson Laboratories #000664) bred in-house were used in this study. While not quantified in the present study, all mice received bilateral injections of cholera toxin b (Invitrogen #C34776) into the lateral septum.

### Auditory Threat Conditioning

Mice were randomly assigned to naïve or trained groups, then acclimated in an airlock adjacent the conditioning room for at least 30 min. Cued threat conditioning was conducted in Habitest modular operant chambers housed within sound-attenuating cubicles (Coulbourn). Conditioning consisted of a 240 s habituation period followed by six co-terminating pairings of the CS (20 s, 2 kHz, 65 dB pure tone) with the US (2 s, 0.5 mA footshock) with 100 s inter-trial intervals. Mice were removed from the operant chamber 40 s after the last CS-US pairing and returned to a holding cage in the airlock. Operant chambers were cleaned with 70% EtOH between animals.

Freezing was quantified with Actimetrics FreezeFrame V4 (Coulbourn). Freezing thresholds were determined for each animal based on the highest movement index value for which the mouse showed no movement except respiration for ≥1 s. Shock reactivity was estimated by the FreezeFrame motion index (Anagnostaras et al., 2010). Instances of darting, defined as a continuous rapid motion across the operant chamber, were manually scored.

### Immunofluorescence Staining

Mice were anesthetized and transcardially perfused with phosphate buffered saline (PBS) and 4% paraformaldehyde 90 min after exiting the operant chamber. Brains were postfixed overnight in 4% paraformaldehyde, washed with PBS, cryoprotected in 30% sucrose, embedded in a 2:1 mixture of O.C.T. Compound (Thermo Fisher Scientific #23730571) to 30% sucrose, and stored at –80°C until sectioning. Serial coronal sections (50 μm thick in 300 μm intervals) were obtained on a cryostat.

Immunofluorescence staining was conducted as previously described (Lucas et al., 2012). Antibodies included rabbit anti-c-fos primary antibody (Synaptic Systems #226003; 0.25 μg/mL dilution) and donkey anti-rabbit 647 secondary antibody (Jackson ImmunoResearch #711605152; 1.25 μg/mL dilution). Sections were counterstained with DAPI (Invitrogen #D3571; 0.2 μg/mL dilution), mounted onto slides, coverslipped with Prolong Gold Antifade Mountant (Life Technologies #P36930), and stored at 4°C until imaging.

Imaging was conducted on an Olympus FV3000 confocal microscope. Brain sections from a trained female were used to set the c-fos laser power, voltage, gain, and offset, and these settings were held constant across all brain sections and animals. Images were acquired at 20x on a single z plane focused at 10 μm tissue depth. For presentation in figures, brightness and contrast were uniformly altered across all images for a given brain region.

### Quantification of c-fos

The semi-automated software package WholeBrain (Furth et al., 2018) was used to quantify immunopositive c-fos nuclei in all brain regions ranging from +2.80 to –3.52 mm from bregma. Detection of c-fos was set to the same soma area threshold and pixel intensity range for all images. Left and right hemisphere counts at the same bregma value were averaged together to represent a single regional value per section, and section values were averaged together to obtain a single value per region. All images were visually inspected for quality control, and regions were excluded from analysis due to damage, imaging errors, or lack of matched representation across animals. The final dataset included 112 brain regions.

### Statistics

Two-tail independent-samples *t*-test, two-way ANOVA, and two-way repeated-measures ANOVA were implemented to determine statistical significance. Planned *posthoc* comparisons maintained familywise error at 0.05.

### Correlation Matrix Construction

Correlation matrices within each experimental group were constructed by calculating Pearson’s correlation coefficient *r* for all pairwise comparisons of c-fos counts between all 112 brain regions.

### Functional Network Construction

Networks of correlated interregional c-fos counts were constructed by thresholding the correlation matrix to create a binary adjacency matrix for each group. We used a significance level of *p* < 0.05, corresponding to Pearson’s correlation coefficient *r* ≥ 0.95 for naïve groups (*n* = 4) and *r* ≥ 0.997 for trained groups (*n* = 3), as the threshold for considering two brain regions functionally connected. We only considered positive correlations. Due to the low *n* per group, we could not adjust *p* values for multiple comparisons without eliminating most connections, so a high false positive rate may exist among connections in the networks. Random networks were constructed by shuffling connections in the data-generated networks to assess whether the measures found in the data-generated networks were likely to have been generated through random selection of false positives or the chosen *p*-value threshold (Rubinov and Sporns, 2010).

### Functional Network Analysis

Analysis of the functional networks created from interregional correlation matrices generally followed the approach taken by Wheeler et al. (2013). Small-worldness was calculated as the ratio of transitivity to the average shortest path length, whereby both are normalized to the same measures computed for 100 Erdös-Rényi random graphs (Humphries and Gurney, 2008). Transitivity, assortativity, node degree, and betweenness were all calculated using functions from the Brain Connectivity Toolbox (Rubinov and Sporns, 2010). We used Markov clustering to determine cluster membership for each node, setting the inflation hyperparameter independently for each graph by finding the value that maximized a measure of modularity across the network (Malliaros and Vazirgiannis, 2013).

All measures were computed in Python using a combination of the Python implementation^1^ of the Brain Connectivity Toolbox (Rubinov and Sporns, 2010), the Python implementation^2^ of the Markov clustering algorithm^3^, and the networkx Python package. All code used for the network analysis and visualization are available on Github.

## Data Availability Statement

The raw data supporting the conclusions of this article will be made available by the authors, without undue reservation.

## Ethics Statement

The animal study was reviewed and approved by the Institutional Animal Care and Use Committee at North Carolina State University.

## Author Contributions

EL did the conceptualization, carried out the resources, and supervised the data. KD, SB, TR, and EL performed the methodology, investigated the data, and wrote, reviewed, and edited the manuscript. KD, TR, and EL wrote the original draft. KD and EL carried out the funding acquisition. All authors contributed to the article and approved the submitted version.

## Conflict of Interest

TR is an employee of IBM Research. The remaining authors declare that the research was conducted in the absence of any commercial or financial relationships that could be construed as a potential conflict of interest.

## Publisher’s Note

All claims expressed in this article are solely those of the authors and do not necessarily represent those of their affiliated organizations, or those of the publisher, the editors and the reviewers. Any product that may be evaluated in this article, or claim that may be made by its manufacturer, is not guaranteed or endorsed by the publisher.

## Footnotes

1
https://github.com/aestrivex/bctpy

2
https://github.com/guyallard/markov_clustering

3
https://micans.org/mcl
