# Supplementary material for: Sex-Specific Neural Networks of Cued Threat Conditioning: A Pilot Study
Source: Front Syst Neurosci. 2022 May 17;16:832484. doi: 10.3389/fnsys.2022.832484 (PMC9152023; doi:10.3389/fnsys.2022.832484)
Supplement: Supplementary file 1 [file Data_Sheet_1.pdf]

Supplementary Material

1. Supplementary Methods

1.1 Animals

All experiments were approved in advance by the Institutional Animal Care and Use Committee at NC State University and conducted in accordance with the National Institutes of Health Guide for the Care and Use of Laboratory Animals. Adult (8-12 weeks) male and female C57BL/6J mice (Jackson Laboratories, Bar Harbor, ME, USA; #000664) bred in-house were used in this study. Mice were housed 2-4 per cage with *ad libitum* access to food and water and maintained on a 12-hour light/dark cycle (lights on at 8:00 AM) in an environmentally controlled husbandry room. While not quantified in the current study, all mice received bilateral stereotaxic infusions of the retrograde tracer cholera toxin B (0.35 uL; Invitrogen, Waltham, MA, USA; #C34776) into the lateral septum (mm from bregma: AP +0.6, ML ± 0.38, DV -3.2) under isoflurane anesthesia, followed by three days of analgesia with carprofen (5 mg/kg, s.c.). Cued threat conditioning was conducted seven days after surgery. The initial n/group consisted of 5 naïve and 10 trained animals per sex. However, animals were excluded from downstream analysis for the following reasons: surgical mistargeting (4 trained female, 3 trained male), poor perfusion (2 trained female, 1 trained male), perfused at wrong time (2 trained male), and behavioral software malfunction (1 trained male). Unfortunately, the COVID-19 campus shutdown prevented the inclusion of additional cohorts of animals to this experiment.

1.2 Auditory Threat Conditioning

All mice were tested by the same researcher (S.B.) between 9:00-10:30 AM. Male and female mice were tested in separate cohorts. Mice were randomly assigned to naïve or trained groups, then acclimated in an airlock outside the conditioning room for at least 30 min. Cued threat conditioning was conducted in Habitest modular operant chambers (17.78 cm x 17.78 cm x 30.48 cm) housed within sound-attenuating cubicles (Coulbourn, Holliston, MA, USA). A near infrared camera was mounted at a 45° angle behind each operant chamber, and Actimetrics FreezeFrame4 software (Coulbourne) provided automated stimulus delivery and recording of behavioral sessions. Cued threat conditioning consisted of a 240 s habituation period followed by six co-terminating pairings of the CS (20 s, 2 kHz, 65 dB pure tone) with the US (2 s, 0.5 mA footshock) with 100 s inter-trial intervals. Mice were removed from the operant chamber 40 s after the last CS-US pairing and returned to a holding cage in the airlock. Operant chambers were cleaned with 70% EtOH between animals.

Freezing was quantified offline with Actimetrics FreezeFrame V4. Freezing thresholds were determined for each animal based on the highest movement index value for which the mouse showed no movement except respiration for ≥ 1 s. Shock reactivity was assessed by the FreezeFrame motion index, which measures displacement of pixels between video frames captured at 3.75 Hz to estimate animal movement (Anagnostaras et al., 2010). Instances of darting, defined as a continuous rapid motion across the operant chamber, were manually scored.

1.3 Immunofluorescence Staining

Mice were anesthetized with avertin (i.p.; 240 mg/kg) prior to transcardial perfusion with phosphate buffered saline (PBS) and 4% paraformaldehyde 90 min after exiting the operant chamber. Anesthesia and perfusion of naïve and trained mice was interleaved to account for order and time-of-day effects. Following decapitation and removal, brains were postfixed overnight in 4% paraformaldehyde, washed with PBS, cryoprotected in 30% sucrose, and stored at 4°C for 1-4 days. Brains were then embedded in a 2:1 mixture of O.C.T. Compound (Fisher Scientific, Waltham, MA, USA; #23730571) to 30% sucrose and stored at -80°C until sectioning. Serial coronal sections (50 µm thick in 300 µm intervals) were obtained on a cryostat. Sections were washed with PBS and stored in 60% glycerol at -20°C.

Immunofluorescence staining was conducted as previously described (Lucas et al., 2014; Lucas et al., 2012). Briefly, sections were washed in PBS and blocked in 10% donkey serum prior to incubation in rabbit anti-c-fos primary antibody (Synaptic Systems, Goettingen, Germany; #226003; 0.25 µg/mL dilution) with 5% donkey serum for 48 hrs at 4°C. After washes with PBS, sections were incubated in donkey anti-rabbit 647 secondary antibody (Jackson ImmunoResearch, West Grove, PA, USA; #711605152; 1.25 µg/mL dilution) with 5% donkey serum for 2 hrs at room temperature. Sections were washed with PBS, counterstained with DAPI (Invitrogen #D3571; 0.2 µg/mL dilution), mounted onto non-charged microscope slides, coverslipped with Prolong Gold Antifade Mountant (Life Technologies, Carlsbad, CA, USA; #P36930), and stored at 4°C until imaging.

Stained sections were imaged on an upright Olympus FV3000 confocal microscope equipped with solid-state lasers (Olympus, Waltham, PA). Brain sections from a trained female were used to set the c-fos laser power, voltage, gain, and offset, and these settings were held constant across all brain sections and animals. Tiled images for each brain section were acquired at 20x on a single z plane focused at 10 µm tissue depth. For presentation in figures, brightness and contrast were uniformly altered across all images for a given brain region.

1.4 Quantification of c-fos

All images were analyzed blind to the experimental group by the same experimenter (K.C.D.). The semi-automated software package WholeBrain (v0.1.35; Furth et al., 2018) was used to quantify immunopositive c-fos nuclei in all brain regions ranging from +2.80 to -3.52 mm from bregma. Flat-field correction and stitching of image tiles produced grayscale 8-bit TIF images of brain sections. Detection of c-fos was set to the same soma area threshold and pixel intensity range for all tiled images. WholeBrain-generated outlines corresponding to the Allen Mouse Brain Atlas were registered, overlaid, and manually adjusted for each brain section. Left and right hemisphere counts at the same bregma value were averaged together to represent a single regional value per section, and section values were averaged together to obtain a single value per region. All images were visually inspected for quality control, and regions were excluded from analysis due to damage, imaging errors, or lack of matched representation across animals. The final dataset included 112 brain regions. Three brains were missing c-fos counts from specific regions due to sectioning or imaging errors and were therefore excluded from the affected regional analysis and eliminated from all interregional analyses.

1.5 Statistics

Behavioral and regional c-fos expression data were analyzed with Graphpad Prism 7.04 (La Jolla, CA, USA) or IBM SPSS Statistics 26 (Armonk, NY, USA), and statistical significance was set at 0.05. Freezing, shock reactivity, and darting during threat conditioning was analyzed by two-way repeated-measures ANOVA to compare behavior across CS-US pairings between sexes. For freezing during inter-trial intervals, the sphericity assumption was violated, and statistical values are thus reported from the Greenhouse-Geisser correction. Shock reactivity was also averaged across US presentations and analyzed with a two-tailed independent-samples t-test. Two-way ANOVA was used to compare regional c-fos expression by sex and behavioral condition. In the case of a significant interaction, Fisher's LSD was used to conduct planned posthoc comparisons between trained males and trained females. While potentially interesting, no other posthoc comparisons were made in order to retain familywise α at 0.05.

1.6 Correlation matrix construction

Correlation matrices within each experimental group were constructed by calculating Pearson's correlation coefficient r for all pairwise comparisons of c-fos counts between all 112 brain regions.

1.7 Functional network construction

Networks of correlated interregional c-fos counts were constructed by thresholding the correlation matrix to create a binary adjacency matrix for each group. We used a significance level of $p < 0.05$, corresponding to Pearson's correlation coefficient $r \geq 0.95$ for naïve groups (n=4), and $r \geq 0.997$ for trained groups (n=3), as the threshold for considering two brain regions functionally connected. We only considered positive correlations, consistent with related studies performing similar computational analyses (Tanimizu et al., 2018; Vetere et al., 2017; Wheeler et al., 2013). Due to the low n per group, we could not adjust p values for multiple comparisons without eliminating most connections, so a high false positive rate may exist among connections in the networks. To establish controls that could indicate whether identified differences in data-generated networks were a result of the potential inclusion of false positive connections, we constructed random networks for every data-generated network that we analyzed. Random networks were constructed by shuffling connections in the data-generated networks using the 'randomizer_bin_und' function from the Python implementation (https://github.com/aestrivex/bctpy) of the Brain Connectivity Toolbox (Rubinov and Sporns, 2010), which maintains the number of active nodes (with at least 1 neighbor), total number of edges, and degree distribution (number of edges per node). The random networks were therefore appropriately matched to the data-generated networks in local properties. We also compared data-generated and random networks across 20 different p-value thresholds to establish that network properties did not depend on the chosen threshold.

1.8 Functional network analysis

Analysis of the functional networks created from interregional correlation matrices generally followed the approach taken by Wheeler et al. (2013). Several measures were used to quantify the structure present in the functional networks. *Transitivity*, the probability that two nodes each connected to a third node will also be connected to each other, measures network segregation (Newman, 2003). *Assortativity* measures the correlation between degrees of nodes at either end of a connection, with positive values indicating that nodes tend to link to other nodes with similar degree

(Newman, 2002). *Small-worldness* quantifies the extent to which a network has a small-world structure with both a high level of local clustering and short paths across the network between clusters. Small-worldness was calculated as the ratio of transitivity to the average shortest path length, whereby both are normalized to the same measures computed for 100 Erdös-Rényi random graphs (Humphries and Gurney, 2008). Transitivity, assortativity, node degree, and betweenness were all calculated using functions from the Brain Connectivity Toolbox (Rubinov and Sporns, 2010). We used Markov clustering to determine cluster membership for each node, setting the inflation hyperparameter independently for each graph by finding the value that maximized a measure of modularity across the network (Malliaros and Vazirgiannis, 2013).

All measures were computed in Python using a combination of the Python implementation (https://github.com/aestrivex/bctpy) of the Brain Connectivity Toolbox (Rubinov and Sporns, 2010), the Python implementation (https://github.com/guyallard/markov_clustering) of the Markov clustering algorithm (https://micans.org/mcl), and the networkx Python package. All code used for the network analysis and visualization are available on Github.

2. List of Supplementary Tables

Supplementary Table 1. ***List of brain regions and acronyms.***

Supplementary Table 2. ***Group data and statistical analyses of regional c-fos expression.***

Supplementary Table 3. ***Ordered list of brain regions for cross correlation matrices in Figure 3.***

3. Supplementary Figures

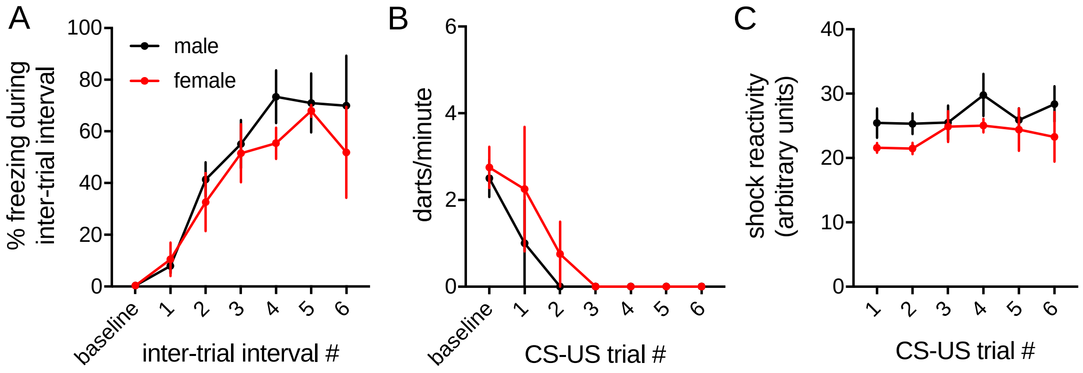

Supplementary Figure 1. ***Extended analysis of behavior during cued threat conditioning.*** **(A)** No sex differences in freezing during the inter-trial intervals between CS-US pairings. Two-way repeated-measures ANOVA, main effect of CS-US pairing ($F_{(1.76,\ 8.78)} = 15.36$, $p = 0.002$), no main effect of sex ($p = 0.50$), and no interaction ($p = 0.58$). **(B)** No sex differences in darting during the baseline period or during the CS across CS-US pairings. Two-way repeated-measures ANOVA, main effect of CS-US pairing ($F_{(6,30)} = 7.43$, $p < 0.0001$), no main effect of sex ($p = 0.48$), and no interaction ($p = 0.87$). No darting was observed during the inter-trial intervals. **(C)** No sex differences in shock reactivity during the US across CS-US pairings. Two-way repeated-measures ANOVA, no main effect of CS-US pairing ($p = 0.40$), no main effect of sex ($p = 0.21$), and no interaction ($p = 0.86$).

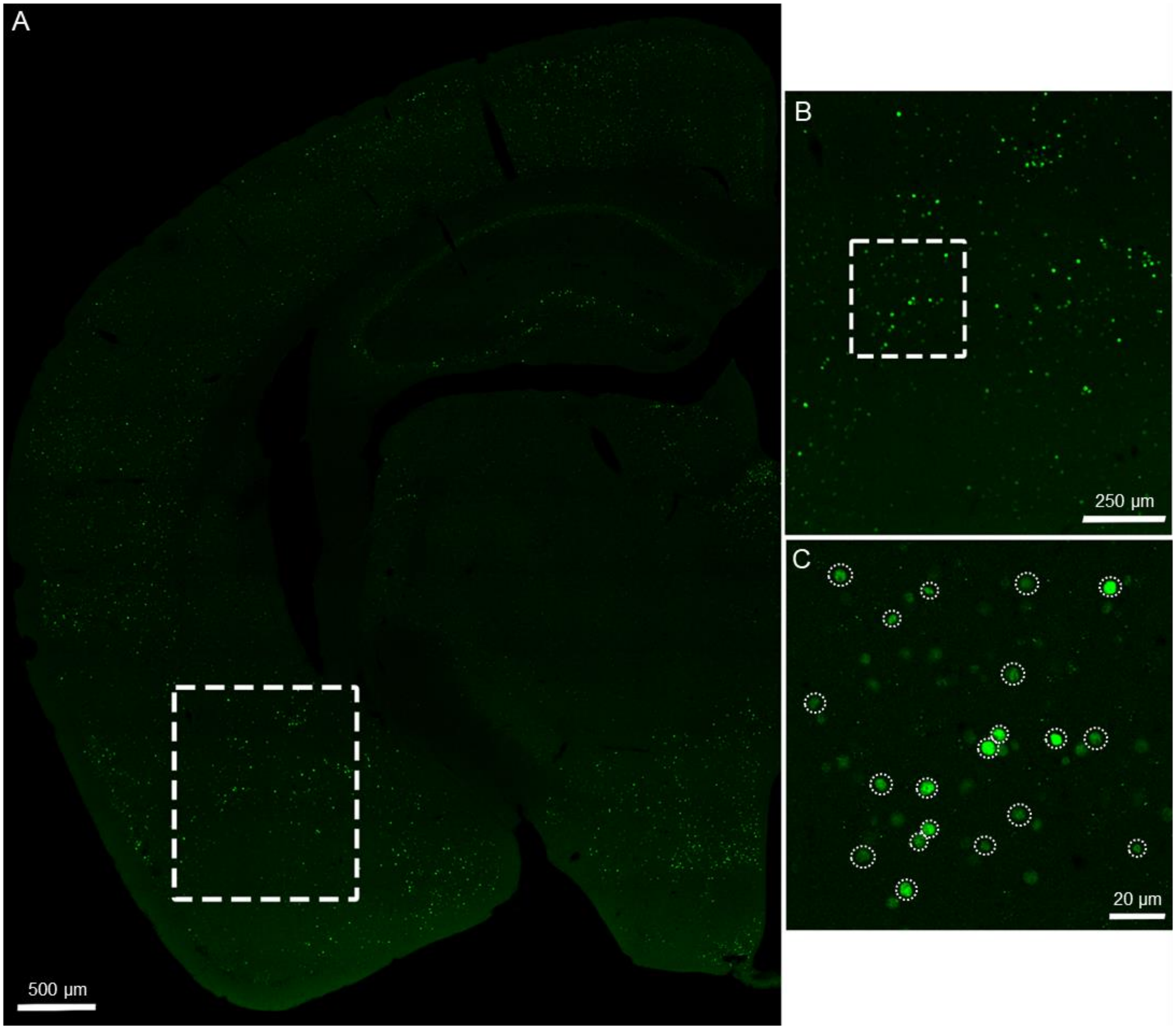

Supplementary Figure 2. ***Extended representation of confocal microscopy of c-fos immunolabeling.*** Tiled images of coronal brain sections were obtained at 20x on a single z-plane for quantification of c-fos immunopositive nuclei with the semi-automated program WholeBrain (Furth et al., 2018). A representative image used in the experimental pipeline is shown. The image has been cropped to display one hemisphere for the purpose of presentation in this figure. No other adjustments have been made. The boxed portion of the image in **(A)** contains the basolateral amygdala, shown at higher magnification in **(B)**. The boxed portion of the image in B contains the lateral nucleus of the basolateral amygdala, shown at higher magnification in **(C).** Immunopositive cells segmented by WholeBrain and included in downstream analyses are circled in C.

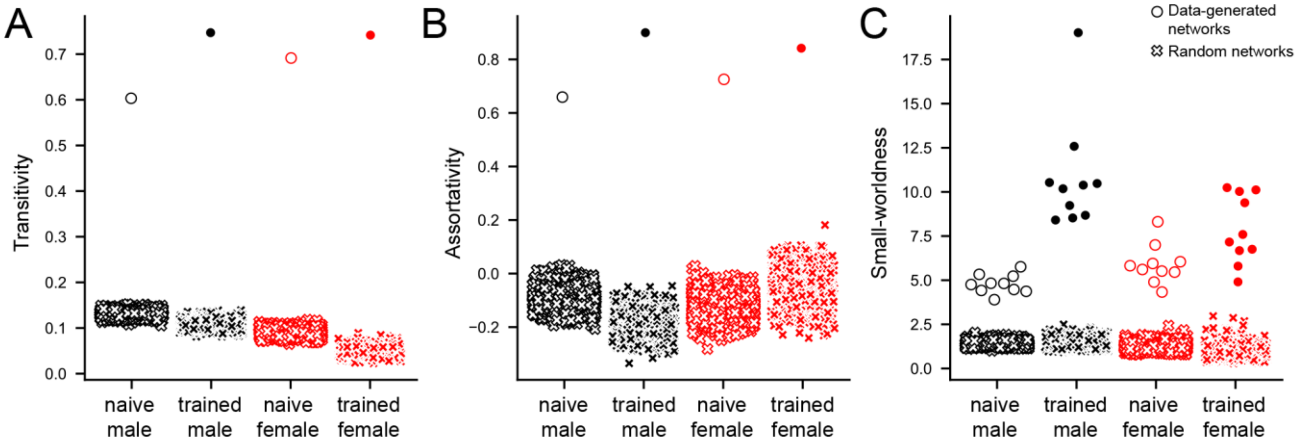

Supplementary Figure 3. ***Comparison of the functional structure of data-generated versus random networks.*** Network-level graph-theoretic measures of **(A)** transitivity, **(B)** assortativity, and **(C)** small-worldness were compared between data-generated functional networks (circles) and random networks (X's). Data-generated networks were created using a correlation significance threshold of $p < 0.05$, and 1000 random networks were generated by shuffling the supra-threshold connections present in each data-generated network while maintaining the degree distribution, number of nodes, and number of edges. Small-worldness was calculated 10 times per group as this measure involves generating random comparison networks.

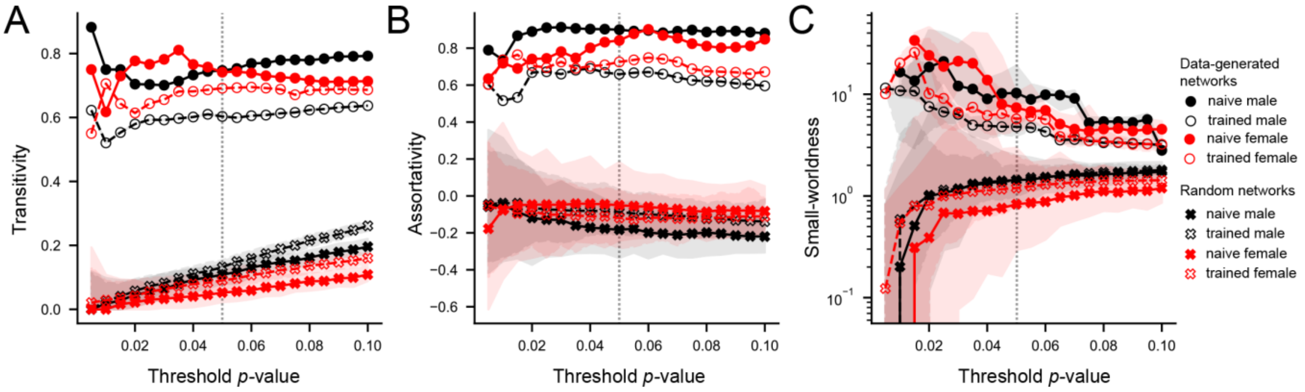

Supplementary Figure 4. ***Analysis of network graph properties across a range of correlation thresholds.*** Network-level graph-theoretic measures of **(A)** transitivity, **(B)** assortativity, and **(C)** small-worldness were compared between data-generated (circles) and random (X's) networks across a range of correlation significance thresholds. 1000 random networks were generated per group, matched to data-generated networks in degree distribution, number of nodes, and number of edges. Small-worldness was calculated 10 times per group as this measure involves generating random comparison networks. Shaded regions show data range (minimum to maximum). Dotted lines demarcate the significance threshold ($p < 0.05$) for data-generated networks in Figure 4.

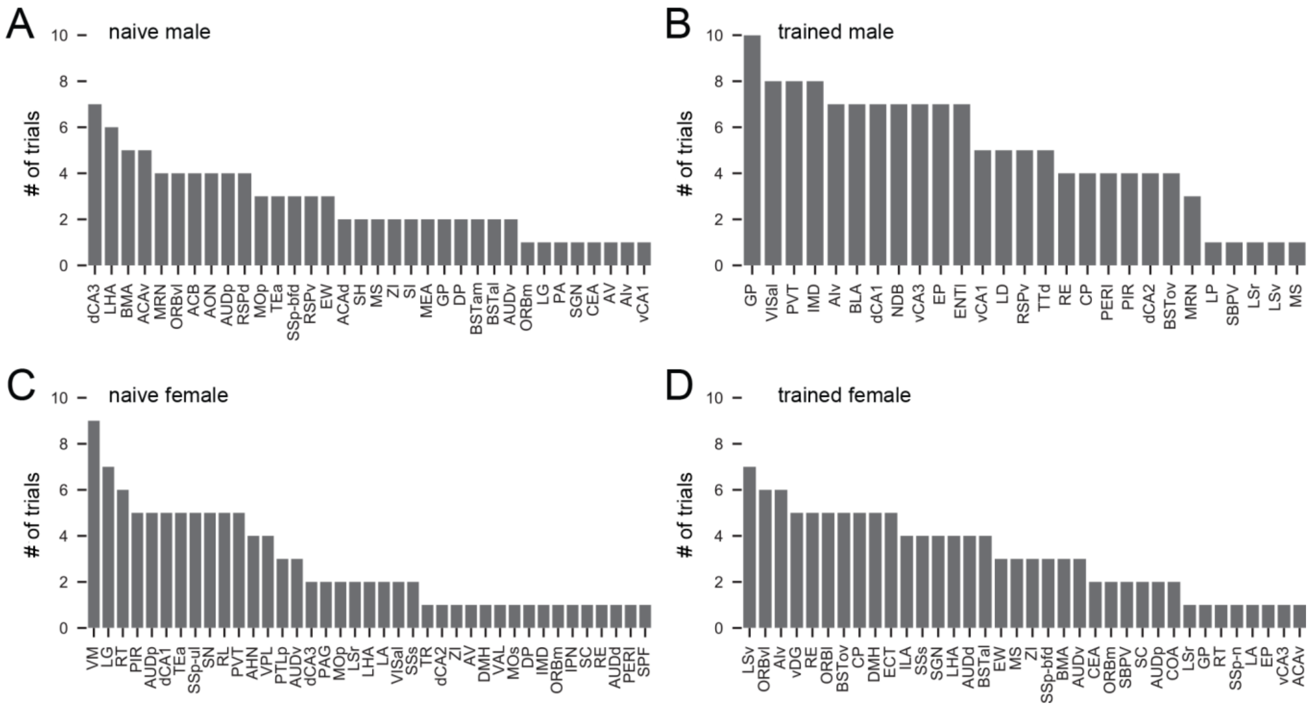

Supplementary Figure 5. ***Threshold pruning reveals robust hub regions.*** Hub region status was calculated at 10 different *p*-value thresholds (0.005 to 0.05) to identify regions that reliably emerge as network hubs across groups. Each graph displays the number of threshold levels for which a region was included as a hub region for **(A)** naïve male, **(B)** trained male, **(C)** naïve female, and **(D)** trained female networks. See Table S1 for a list of regions and their acronyms.

4. References

Anagnostaras, S.G., Wood, S.C., Shuman, T., Cai, D.J., Leduc, A.D., Zurn, K.R., Zurn, J.B., Sage, J.R., and Herrera, G.M. (2010). Automated assessment of pavlovian conditioned freezing and shock reactivity in mice using the video freeze system. *Front Behav Neurosci* 4.

Furth, D., Vaissiere, T., Tzortzi, O., Xuan, Y., Martin, A., Lazaridis, I., Spigolon, G., Fisone, G., Tomer, R., Deisseroth, K., et al. (2018). An interactive framework for whole-brain maps at cellular resolution. *Nat Neurosci* 21, 139-149.

Humphries, M.D., and Gurney, K. (2008). Network 'small-world-ness': a quantitative method for determining canonical network equivalence. *PLoS One* 3, e0002051.

Lucas, E.K., Dougherty, S.E., McMeekin, L.J., Reid, C.S., Dobrunz, L.E., West, A.B., Hablitz, J.J., and Cowell, R.M. (2014). PGC-1alpha provides a transcriptional framework for synchronous neurotransmitter release from parvalbumin-positive interneurons. *J Neurosci* 34, 14375-14387.

Lucas, E.K., Dougherty, S.E., McMeekin, L.J., Trinh, A.T., Reid, C.S., and Cowell, R.M. (2012). Developmental alterations in motor coordination and medium spiny neuron markers in mice lacking PGC-1alpha. *PLoS One* 7, e42878.

Malliaros, F.D., and Vazirgiannis, M. (2013). Clustering and community detection in directed networks: A survey. *Physics Reports* 533, 95-142.

Newman, M.E. (2002). Assortative mixing in networks. *Phys Rev Lett* 89, 208701.

Newman, M.E.J. (2003). The structure and function of complex networks. *SIAM Review* 45, 167-256.

Rubinov, M., and Sporns, O. (2010). Complex network measures of brain connectivity: uses and interpretations. *Neuroimage* 52, 1059-1069.

Tanimizu, T., Kono, K., and Kida, S. (2018). Brain networks activated to form object recognition memory. *Brain Res Bull* 141, 27-34.

Vetere, G., Kenney, J.W., Tran, L.M., Xia, F., Steadman, P.E., Parkinson, J., Josselyn, S.A., and Frankland, P.W. (2017). Chemogenetic interrogation of a brain-wide fear memory network in mice. *Neuron* 94, 363-374 e364.

Wheeler, A.L., Teixeira, C.M., Wang, A.H., Xiong, X., Kovacevic, N., Lerch, J.P., McIntosh, A.R., Parkinson, J., and Frankland, P.W. (2013). Identification of a functional connectome for long-term fear memory in mice. *PLoS Comput Biol* 9, e1002853.
